# Epigenetically Mediated Ciliogenesis and Cell Cycle Regulation, and Their Translational Potential

**DOI:** 10.3390/cells10071662

**Published:** 2021-07-02

**Authors:** Linda Xiaoyan Li, Xiaogang Li

**Affiliations:** 1 Department of Internal Medicine, Mayo Clinic, Rochester, MN 55905, USA; li.xiaoyan@mayo.edu; 2 Department of Biochemistry and Molecular Biology, Mayo Clinic, Rochester, MN 55905, USA

**Keywords:** cell cycle, primary cilia, epigenetic regulator, ciliogenesis

## Abstract

Primary cilia biogenesis has been closely associated with cell cycle progression. Cilia assemble when cells exit the cell cycle and enter a quiescent stage at the post-mitosis phase, and disassemble before cells re-enter a new cell cycle. Studies have focused on how the cell cycle coordinates with the cilia assembly/disassembly process, and whether and how cilia biogenesis affects the cell cycle. Appropriate regulation of the functions and/or expressions of ciliary and cell-cycle-associated proteins is pivotal to maintaining bodily homeostasis. Epigenetic mechanisms, including DNA methylation and histone/chromatin modifications, are involved in the regulation of cell cycle progression and cilia biogenesis. In this review, first, we discuss how epigenetic mechanisms regulate cell cycle progression and cilia biogenesis through the regulation of DNA methylation and chromatin structures, to either promote or repress the transcription of genes associated with those processes and the modification of cytoskeleton network, including microtubule and actin. Next, we discuss the crosstalk between the cell cycle and ciliogenesis, and the involvement of epigenetic regulators in this process. In addition, we discuss cilia-dependent signaling pathways in cell cycle regulation. Understanding the mechanisms of how epigenetic regulators contribute to abnormal cell cycle regulation and ciliogenesis defects would lead to developing therapeutic strategies for the treatment of a wide variety of diseases, such as cancers, polycystic kidney disease (PKD), and other ciliopathy-associated disorders.

## 1. Introduction

The cell cycle is a complicated and finely tuned process that takes place in a cell as it grows and divides, and is composed of interphase (G_1_, S, and G_2_ phases), followed by the mitotic phase (mitosis and cytokinesis), and the G_0_ (quiescence) phase [1,2]. There are at least two decisive cell cycle points identified in the G_1_ and G_2_ phases, whereas the G_0_/G_1_ phases seem more variable in different cells, resulting in the variable length of the cell cycle [3]. The regulation of the cell cycle and cell proliferation rates varies in different cells.

**Figure 1:**
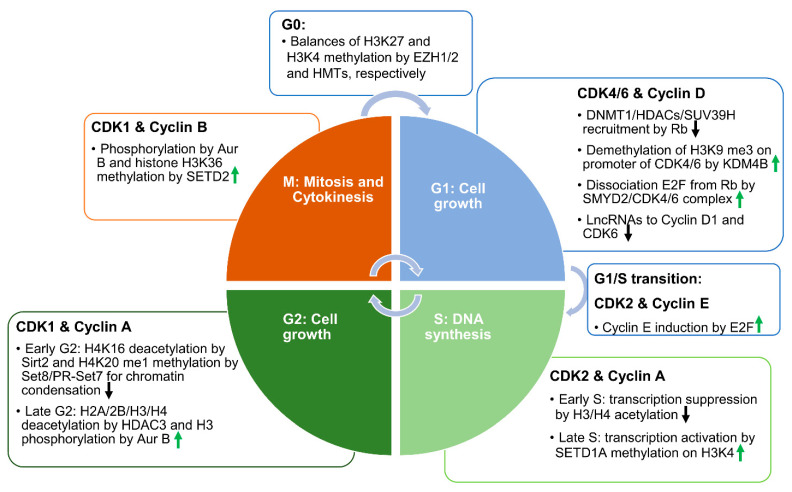
The associations between CDKs, cyclins, and the epigenetic mechanisms in each phase of the cell cycle. Schematic diagram showing the functions of different CDK–cyclin pairs in each phase of the cell cycle. In the G_1_ phase, CDK4 or CDK6 coupled with cyclin D phosphorylates and removes Rb from the Rb–E2F complex to facilitate E2F-mediated transcription of cell cycle effectors. CDK4 and CDK6 interact with a histone methyltransferase—SMYD2—to regulate its activity. SMYD2 methylation of Rb may facilitate the phosphorylation of Rb by CDK4/6. In addition, SMYD2 directly regulates the transcription of CDK4 and CDK6. In the S phase, CDK2 coupled with cyclin A or E reinforces the phosphorylation of Rb to promote DNA synthesis. In the early S phase, the acetylation of histones suppresses transcription, while later in the S phase, SETD1A-mediated histone methylation reactivates transcription, and promotes the transition from the S phase to the G_2_ phase. In the G_2_ phase, CDK1 coupled with cyclin A or B phosphorylates histone tails at different sites to promote mitotic entry. HDAC3-mediated histone deacetylation is involved in this process. In the meantime, SIRT2-mediated deacetylation of histone H4K16 and SET8/PR-SET7-mediated methylation of H4K20 synergistically regulate cell cycle progression. Eventually, when histone H3Ser10 is globally phosphorylated, cells enter the mitosis phase. In the M phase, SETD2-mediated tubulin and histone methylation promotes cytokinesis and the separation of cells into two daughter cells. In the G_0_ phase, histone methyltransferase (HMT)-mediated H3K4 trimethylation and EZH1/2 -mediated H3K27 trimethylation dynamically control the chromatin status. Upward-pointing green arrows represent the propelling effects on the cell cycle. Downward-pointing black arrows represent the inhibiting effects on the cell cycle.

The cell cycle is regulated by numerous regulatory proteins, which direct a cell through a series of highly ordered events, culminating in the production of two daughter cells. The central regulators of the cell cycle are cyclin-dependent kinases (CDKs), which form complexes with the corresponding cyclins in different phases to promote cell cycle progression (Figure 1) [4,5,6,7]. The activity of CDKs can be inhibited by their canonical inhibitors—including p16, p21, and p27—via direct interaction with CDK–cyclin complexes [8,9]. Recent studies indicate that epigenetic mechanisms are involved in the regulation of cell cycle progression and the expression of CDKs (Figure 1).

The cilium is a cell-cycle-associated organelle, in that cilia formation is dynamically coordinated with cell cycle progression. The cilium initiates its assembly at the G_0_ or G_1_ phase and elongates through the S phase until centrosomes commence duplication, at which point cilia resorption progresses to completion before the cells begin mitosis. After mitosis, when cells re-enter the cell cycle, centrosomes are again able to assemble primary cilia at either the G_0_ or early G_1_ phase [10,11]. Factors that repress the transition from the late M phase to the G_1_ phase or G_1_/S entry—such as the inhibitors of CDK2, CDK4, CDK6, and their corresponding cyclins—would induce the initiation of cilia. In contrast, factors that promote cell cycle to restart from the post-M phase would inhibit cilia formation [12,13]. Thus, the canonical understanding is that the cell cycle plays a predominant role in the regulation of cilia biogenesis. However, recent studies indicate that ciliary assembly proteins can also regulate cell cycle progression, thereby shedding light on the effects of ciliogenesis on cell cycle progression. To further understand the relationship between the cell cycle and ciliogenesis, it is necessary to clarify how the cell cycle and ciliogenesis communicate with one another, and coordinate their processes to an appropriate degree in order to maintain cellular hemostasis.

Recent studies suggest that the cell cycle and cilia biogenesis can be regulated by epigenetic mechanisms [12,14]. Epigenetics is the study of heritable genome-wide changes in phenotype or gene expression caused by mechanisms other than changes in the underlying DNA sequence [15]. Epigenetic mechanisms include DNA methylation, histone modifications, and noncoding-RNA-associated silencing [16]. Growing evidence indicates that there is a well-organized network among cell cycle, ciliogenesis, and epigenetic regulators, in which epigenetic regulators are not only involved in the regulation of the cell cycle and ciliogenesis, but can in turn be regulated by cell cycle regulators and contribute to bodily homeostasis. This review will mainly focus on the current understanding of epigenetic mechanisms in the regulation of the cell cycle and ciliogenesis. In particular, we will focus on how epigenetic mechanisms regulate the crosstalk between the cell cycle and ciliogenesis. In addition, we will discuss the roles and mechanisms of cilia-dependent signaling pathways in cell cycle regulation.

## 2. Epigenetic Regulation on Cell Cycle

The process of cell division and cell proliferation is important for the growth and development of an organism, and organs undergo cellular evolution, renewal, or repair [17,18]. To maintain bodily homeostasis, it is essential to sequentially control the molecular events that occur during the cell cycle. Dysregulation of these processes can lead to various diseases, including cancer and developmental deficiency. The mammalian cell cycle is composed of four phases: G_1_ (the post-mitotic interphase), S (the DNA synthetic phase), G_2_ (the post-synthetic phase), and M (the mitotic phase) [19]. The G_0_ phase is usually difficult to distinguish from G_1_, for which reason it is usually termed the “G_0_/G_1_” phase. The cell cycle is regulated by CDKs and their associated cyclins at different stages (Figure 1). Specifically, CDK4/6 complexes with cyclin D at the early G_1_ phase; CDK2 complexes with cyclin E during the late G_1_ phase, or the G_1_/S phase transition, and complexes with cyclin A at the S phase; CDK1 complexes with cyclin A at the G_2_ phase and cyclin B at the M phase [20]. CDKs can be regulated by the intrinsic regulators, including p53, p21, p16, and cdc25. Epigenetic mechanisms play essential roles in cell cycle regulation, either through controlling the expression of cell-cycle-related genes—such as CDKs—or through the modification of chromatin condensation and controlling the related signaling proteins via histone marks (Figure 1).

### 2.1. Epigenetic Mechanisms in the Regulation of CDKs and Cyclins at Different Stages of the Cell Cycle

Different epigenetic mechanisms are involved in the regulation of cell cycle regulators, including CDKs, cyclins, CDK inhibitors, and other cell-cycle-associated factors—such as Rb and p53. It has been reported that histone deacetylases (HDACs) play important roles in cell cycle regulation by regulating cyclins. HDAC3 directly interacts with cyclin A and regulates cyclin A stability by modulating its acetylation. Because cyclin A is crucial for S phase progression and M phase entry, knockdown of HDAC3 results in the accumulation of cells in the S and G_2_/M phases [21]. HDAC10 also contributes to the cell cycle via modulation of cyclin A2. HDAC10 regulates the expression of let-7 family target HMGA2 via the deacetylation of histone H3 near the let-7 promoter. In HDAC10 knockdown cells, HMGA2 is downregulated, resulting in an increase in the transcriptional repressor E4F at the cyclin A2 promoter, decreasing the expression of cyclin A2 and leading to G_2_/M transition arrest [22]. HDAC1 and HDAC2 were found to be involved in the G_2_/M transition by directly binding to the promoter regions of CDK inhibitors, such as p21, p27, and p57 [23]. Loss of HDAC1 and HDAC2 induces the expression of these genes, leading to a cell cycle block. HDAC6 was reported to promote the growth of glioblastoma cells through downregulating the expression of p21 mediated by TGF-β-SMAD2 signaling [24].

Histone demethylases—such as KDM4A, KDM4B, and KDM4C—regulate cell cycle progression mainly through targeting histone H3K9 methylation. It has been reported that KDM4B/JMJD2B are highly expressed in estrogen receptor (ER)-positive breast cancer, in which KDM4B can bind to the ER, then demethylate repressive histone mark H3K9me3 and recruit members of the SWI/SNF-B and MLL2 chromatin-remodeling complexes to induce gene expression in an estrogen-dependent manner. KDM4B and MLL2 synergistically regulate cancer cell proliferation through their target genes, including myeloblastosis (MYB), myelocytomatosis (MYC), and cyclin D1 (CCND1) [25,26]. In addition, KDM4B was reported to regulate the expression of CDK6 via the demethylation of histone H3K9 on its promoter [25]. Furthermore, the histone demethylase MYC-induced nuclear antigen (MINA), which is highly expressed in many cancers, could positively regulate the expression of cyclins B1, D1, E2, CDK1, CDK2, and CDK4 by directly demethylating H3K9me3 [27].

Aside from DNA methylation and histone or chromatin modifications, long non-coding RNAs (lncRNAs) exert their roles as epigenetic regulators, transcription factors, and post-transcription regulators, and are involved in the development, proliferation, and differentiation of various tissues [28]. Recent studies indicate that lncRNAs can regulate the cell cycle through the modification of cell cycle regulators. For example, in response to DNA damage, the lncRNA for cyclin D1—NcRNA CCND1—functions as a transcription factor to negatively regulate cyclin D1 by inhibiting the activity of the coactivator CBP/p300 at the CCND1 promoter, resulting in cell cycle arrest at the G_1_ phase [29]. Growth-arrested DNA-damage-inducible gene 7 (Gadd7)—another DNA-damage-induced lncRNA—negatively regulates CDK6 expression by dissociating CDK6 mRNA from its stabilizer protein transactive response DNA-binding protein 43 kDa (TDP-43), resulting in the degradation of CDK6 mRNA and the inhibition of G_1_/S transition [30]. Metastasis-associated lung adenocarcinoma transcript 1 (MALAT1) and steroid receptor RNA activator (SRA) are also well-studied lncRNAs, which regulate the expression of p53, p16, and p21 to eventually affect cell cycle progression [31].

### 2.2. Epigenetic Mechanisms in the Regulation of Cell-Cycle-Associated Transcriptional Factors and Chromatin

The role of Rb–E2F signaling in G_1_/S transition has been widely studied. In general, unphosphorylated Rb (active) binds to E2F and inhibits the E2F-mediated expression of genes associated with G_1_/S transition and S phase progression, such as E-type cyclins. Unphosphorylated Rb also recruits HDACs to the promoters of specific cell cycle genes [32] in order to repress the transcription of those genes. In addition, Rb can recruit DNMT1—a DNA methyltransferase—and SUV39H1—a histone methyltransferase (HMT)—to E2F-site-containing promoters, such as cyclin A/E and CDK2, to repress the transcription of those genes [16]. The partial phosphorylation of Rb de-represses the Rb-mediated suppression on E2F target genes, resulting in the expression of cyclin E. SMYD2 methylates Rb and increases its phosphorylation [33]. The expression and activity of SMYD2 can be regulated by CDK4/6 [12]. Thus, when cells enter the early G_1_ phase, the cyclin D–CDK4/6 complex may increase the mono-phosphorylation of Rb via SMYD2-mediated methylation. The mono-phosphorylation of Rb partially releases its inhibition of E2F, leading to the increase in expression of E2F target genes—including cyclin E, which activates CDK2 to further phosphorylate Rb [34]. Hyperphosphorylated Rb is completely dissociated from E2F, resulting in the expression of a wide range of E2F target genes, which drive cells to proceed into the S phase [35].

When a cell enters the S phase, the transcription of genes is suppressed during DNA replication and synthesis. The process of DNA replication spans throughout the entire S phase, with some chromosomal domains being replicated in the early S phase, whereas others are replicated in the late S phase [36]. To maintain genome stability during this phase, it is important to maintain a balance between the expression of early- and late-replicated genes. Thus, it is essential to suppress transcription of the early-replicated genes and to restart transcription once the entire DNA replication process is complete. The transcription suppression and transcription reactivation are finely regulated by epigenetic regulators. It has been reported that the newly synthesized histones H3 and H4 are acetylated before they are assembled into nucleosomes. For instance, the new H4 molecules are acetylated at lysine residues 5 and 12, and those acetylation patterns are conserved from yeast to humans. In yeast, the new H3 molecules can be acetylated at lysine 56 to maintain genome stability and suppress transcription during DNA synthesis in the S phase. After that, the acetylated H3K56-mediated transcriptional suppression can be released by trimethylation of H3K4 (H3K4me3) [37].

The G_2_ phase is a short phase with significant protein synthesis—mainly involving the production of microtubules—which is not clearly separated from the M phase. Thus, it is usually merged with the M phase, which includes mitosis and cytokinesis. Mitosis is comprised of a prophase, metaphase, anaphase, and telophase. The global phosphorylation of histones is upregulated during mitosis. For example, histone H3 phosphorylation is low until the late interphase, and spreads throughout the genome until the prophase, which can be used as a mitotic mark (p-H3-Ser10) [38]. The phosphorylation of histone H3 is mediated by Aurora B, which can be facilitated via HDAC3-mediated histone deacetylation [39]. The phosphorylation of histones is usually accompanied by a decrease in the acetylation of histones, including H3, H4, H2A, and H2B [40]. Among those, H4K16Ac has been reported to be responsible for chromatin decondensation, which is regulated by SIRT2 [41]. In addition, the methylation status of histones H3 and H4 is dynamically changed during the cell cycle. The methylation of H4 at lysine 20 (H4K20)—regulated by SET8/PR-Set7 (responsible for monomethylation), SUV4-20H1 (responsible for dimethylation), and SUV4-20H2 (responsible for trimethylation)—is important for the biological processes that ensure genome integrity during the cell cycle. Compared to di- and trimethylation of H4K20, the monomethylation of H4 at lysine 20 (H4K20) is the most variational mark in the cell cycle. High H4K20me1 levels result in a more compact chromatin structure and induce cell cycle arrest at the G_2_ phase, suggesting the importance of maintaining SET8/PR-Set7 levels to avoid chromatin over compaction in this phase [42].

Cells stop dividing in the G_0_ phase, and have many unique signatures, such as the downregulation of genes that are involved in DNA replication and cell cycle progression, and the upregulation of genes that are involved in transcriptional regulation, including forkhead box O3 (FOXO3) and enhancer of zeste homolog 1 (EZH1). It has been reported that the balanced status between H3K4 trimethylation (associated with transcriptional activation) and H3K27 trimethylation (associated with transcriptional repression) is always maintained in the G_0_ phase [43]. The methyltransferases EZH1 and EZH2, which are responsible for the methylation of H3K27, play important roles at this stage. A prolonged G_0_ phase would result in the shutdown of all cyclins, and the assembly of another group of proteins that form the cilium.

Histone demethylases play important roles in regulating the cell cycle by remodeling chromatins through the removal of histone marks. In addition to KDM4B, other KDM4 family members—including KDM4A, 4C, and 4D—also regulate cell proliferation. KDM4A/JMJD2A is able to demethylate both H3K9 and H3K36, and knockdown of KDM4A results in reduced cell proliferation and delays the G_2_/M phase of the cell cycle [44]. KDM4C/JMJD2C is able to demethylate H3K9me3 to promote androgen-dependent growth of prostate cancer cells in androgen-receptor-positive prostate cancers [44]. The other member—KDM4D—was reported as a coactivator of the androgen receptor to regulate the demethylation of mono-, di-, and tri-H3K9 during DNA replication [45]. LSD1 is another important histone demethylase; a genome-wide mapping of LSD1 cellular localization found that LSD1 is extensively overlapped with H3K4me2 across the genomic regions. LSD1 can be recruited to the chromatin of cells in the G_1_/S/G_2_ phases, and can be removed from the chromatin of M-phase cells, suggesting a correlation of LSD1 and cell cycle regulation, in that LSD1 or H3K4me2 alternatively occupy LSD1 genomic regions during cell cycle progression [46].

### 2.3. Epigenetic Mechanisms in the Regulation of the Cell-Cycle-Associated Cytoskeleton

The cytoskeleton, which gives a cell its shape, is always changing, with polymerization and depolymerization to support all cellular functions. There are two main components of the cytoskeleton: actin, and tubulin. Upon polymerization, actin forms thin filaments (the smallest type of filament), while tubulin forms microtubules (the largest type of filament). The actin-based cytoskeleton plays important roles in spindle-mediated segregation. By using the drug latrunculin B to destroy the actin cytoskeleton, Gachet et al. found that the orientation of the spindle was changed, and the cell cycle was delayed [47]. Microtubules, the main component of spindles, are essential for cell division, segregating duplicated chromosomes into two new cells. It has been reported that histone methyltransferase SETD2 dynamically methylates α-tubulin at lysine 40 (K40), which occurs on the spindle during mitosis and the midbody during cytokinesis. Loss of methylation at this site causes genome instability—including micronuclei, polyploidy, multipolar spindle, and cytokinesis defects—which has been confirmed in SETD2-null mouse embryonic fibroblasts. The C-terminal SRI domain of SETD2 is required for the binding and methylation of α-tubulin. The homozygous SRI domain mutation (Setd2SRI) Setd2SRI/SRI was lethal, while mice with heterozygosity for a Setd2 α-tubulin methylation-deficient allele were haploinsufficient, and heterozygous Setd2 SRI/wt mice exhibited an anxiety-like phenotype [48]. In addition, SETD2 also functions as a histone methyltransferase, and methylates histone H3 at lysine36 (K36), which plays important roles in DNA damage and genome stability [49]. Thus, SETD2 plays dual roles in the methylation of α-tubulin and histone H3 to regulate the cell cycle and genome stability [50].

Histone demethylase KDM3A also plays dual roles in regulating the actin-based cytoskeleton. KDM3A could bind to the promoter of actin gene to transcriptionally regulate the expression of actin. KDM3A could also directly bind with actin proteins and function as a mediator of the actin network by forming a gate at the transition zone of the cilia, which controls the access of ciliary proteins to the cilia and regulates ciliogenesis [51]. Another demethylase—KDM2B—also regulates the actin cytoskeleton. Knockdown of KDM2B decreases the polymerization of actin, while overexpression of KDM2B increases the polymerization of actin. KDM2B-mediated actin polymerization is involved in RhoA/B-GTPases signaling [52].

## 3. Epigenetic Regulation on Ciliogenesis

Cilia are microtubule-based structures that protrude from the cell surface. There are two types of cilia—motile, and non-motile cilia—serving motile and sensory functions, respectively [53,54]. In animals, motile cilia are found on limited tissues, such as respiratory epithelium and choroid plexus epithelial cells [55]. Non-motile cilia, also called primary cilia, can be found on nearly every cell type, and function as sensors for mechanical and chemical environmental cues that regulate cellular differentiation or division [56,57]. In this review, we mainly focus on primary cilia, and the term “cilium/cilia” represents the primary cilium/cilia only, unless otherwise specified. In general, the cilium undergoes cycles of assembly and disassembly that are regulated tightly by a complicated protein network, with proteins trafficking from the base to the cilia tip (anterograde), and from the cilia tip back to the cytoplasm (retrograde), mediated by intraflagellar transport (IFT) complexes of the IFT-B and IFT-A families, driven by a kinesin-2 motor in the anterograde direction and a dynein motor in the retrograde direction, respectively [58].

### 3.1. Epigenetic Mechanisms Mediate the Modification of α-Tubulin in Ciliogenesis

**Figure 2:**
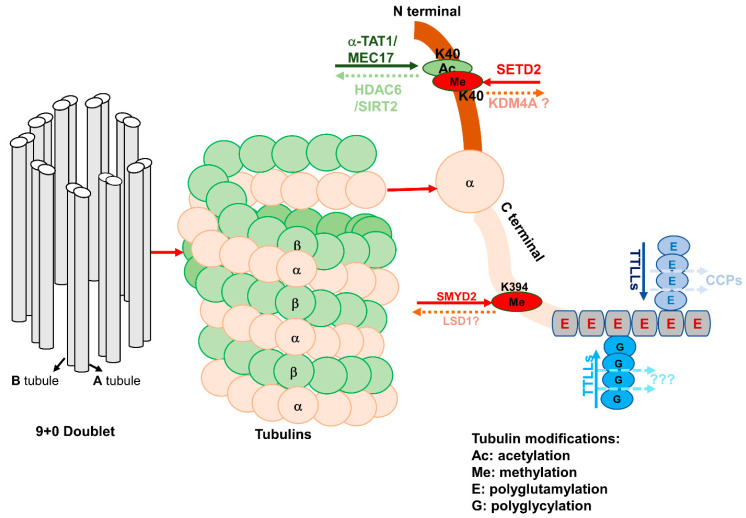
Epigenetic-regulator-mediated tubulin post-translational modifications (PTMs). Left: Schematic drawings of an axoneme composed of 9 doublets (A tubule and B tubule) of microtubules in primary cilia. The microtubules are composed of heterodimers of α-tubulin and β-tubulin. Right: The PTMs of α-tubulin associated with the cell cycle and cilia biogenesis include acetylation, methylation, polyglutamylation, and polyglycylation. Lysine K40 of α-tubulin can be either acetylated by a-TAT1/MEC17 or methylated by SETD2, which can be deacetylated by HDAC6/SIRT2 and possibly demethylated by KDM4A. The acetylation of α-tubulins at K40 occurs on the luminal surface of the microtubules. SMYD2 methylates a-tubulin at K394, which may be demethylated by LSD1. (Poly)glycylation and (poly)glutamylation are found along the glutamate-rich carboxy-terminal tails of α-tubulin, which are regulated by different subsets of tubulin tyrosine ligase-like glutamylases (TTLLs). Ac: Acetylation; Me: methylation; E: polyglutamylation; G: polyglycylation.

At the onset of cilia biogenesis, vesicles dock at the mother centriole and anchor it to the plasma membrane, providing a platform to recruit more proteins and forming the basal body, which serves as a nucleation site for the growth of the axoneme [59]. The microtubule is the core component of the cilia axoneme, and the trafficking of ciliary proteins is microtubule dependent [60]. It has been reported that microtubules are more stable during ciliogenesis [61]. Therefore, any factor that affects microtubule stability would affect the process of ciliogenesis [62,63]. Microtubules are assembled by α-, β-, and γ-tubulin, in which the role of α-tubulin in this process is understood more clearly than others (Figure 2).

**Figure 3:**
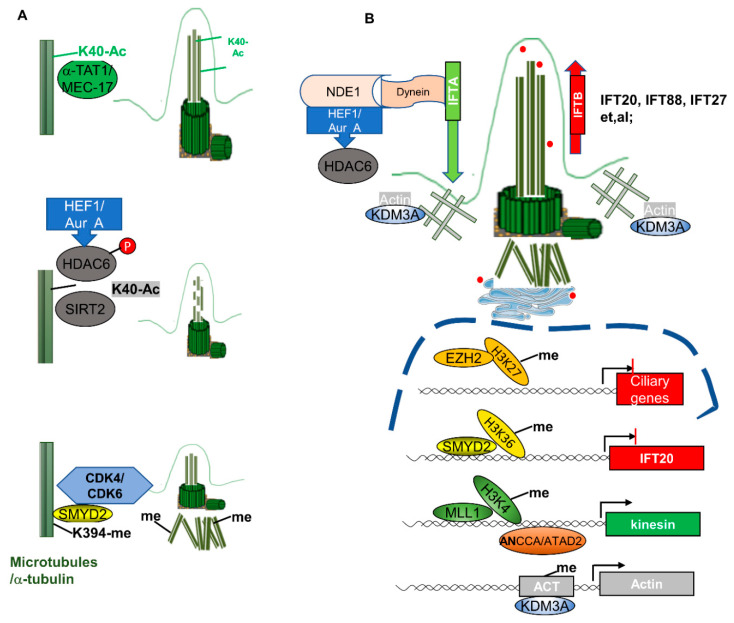
The mechanisms of epigenetic regulators in the regulation of cilia biogenesis. (**A**) Epigenetic regulators regulate cilia biogenesis through modifications of α-tubulin. Top panel: α-TAT1 and MEC-17 mediated the acetylation of α-tubulin at K40, which facilitates the incorporation of α-tubulin into axoneme, promoting cilia assembly. Middle panel: HDAC6- and SIRT2-mediated deacetylation of α-tubulin destabilizes the cilia, resulting in cilia disassembly and short cilia. Bottom panel: SMYD2-mediated methylation of α-tubulin at K394 is accumulated at the basal body, but not in the axoneme, which also facilitates cilia disassembly, resulting in shorter cilia. (**B**) Epigenetic regulators regulate cilia biogenesis by regulating the transcription of ciliary genes. Epigenetic regulators—including EZH2, SMYD2, MLL1, and KDM3A—can regulate the transcription of ciliary genes through modification of histone markers, or by directly binding to the promoters of those genes. For example, KDM3A regulates ciliogenesis by regulating the expression of actin, and by binding to the actin cytoskeleton, creating a responsive “actin gate” that controls the access of IFT proteins to the cilia.

Different modifications—including methylation, acetylation, and glutamylation/glycylation—regulate the stability of α-tubulin (Figure 2). It has been reported that acetylation of α-tubulin at K40 increases its stability. The acetylation of α-tubulin at K40 can be regulated by α-tubulin acetyltransferases, α-TAT1, and its homolog MEC-17 in *C. elegans* [64,65], whereas the deacetylation of α-tubulin at K40 can be regulated by either HDAC6 or SIRT2 [14,66]. By using high-resolution cryo-electron microscopy (cryo-EM) to reconstruct pure samples of αTAT1-acetylated and SIRT2-deacetylated microtubules, it was found that acetylation of αK40 reduces the disorder of the residues of the P37–D47-containing microtubule loop (αK40 loop) and maintains its structure, eventually affecting microtubule stability and function [67]. The acetylation of α-tubulin at K40 by α-TAT1 is required for cilium or flagellum assembly, which was confirmed by siRNAs. Acetylated α-tubulin is more sensitive for cilia assembly compared to these microtubules with unacetylated α-tubulin. Thus, the acetylation of α-tubulin at K40 has been used as a marker to visualize the cilia [68]. Deacetylation of α-tubulin at K40 destabilizes microtubules and induces cilia disassembly [69]. It has been reported that histone deacetylase HDAC6 could remove the acetyl mark from K40 of α-tubulin and induce cilia disassembly. The underlying mechanism is that HEF1-dependent kinase Aur A is activated in response to cellular mitotic signals—such as serum addition—and then phosphorylates and activates HDAC6, which destabilizes the ciliary axoneme and causes cilia disassembly (Figure 3A), causing cells to re-enter the cell cycle [14]. SIRT2 also deacetylates α-tubulin at K40. Knockdown of SIRT2 with siRNA or inhibition of SIRT2 with its inhibitor blocks cilia disassembly, while overexpression of SIRT2 decreases the percentage of ciliated renal epithelial cells [66]. The facts that HDAC6 and SIRT2 deacetylate α-tubulin at the same lysine site (K40), and that SIRT2 can form a complex with HDAC6, suggest that HDAC6 and SIRT2 may work together to regulate ciliogenesis. However, since inhibition of either SIRT2 or HDAC6 alone is sufficient to induce hyperacetylation of α-tubulin and block cilia disassembly, this suggests that they may regulate ciliogenesis independently [66].

α-Tubulin can also be methylated by histone/lysine methyltransferases—another epigenetic regulation on microtubule stability. Recently, we found that histone methyltransferase SMYD2 methylates α-tubulin at K394 [12], resulting in destabilization of microtubules and inhibition of ciliogenesis. Inhibition of SMYD2 with its inhibitor AZ505 or knockdown of SMYD2 with siRNA promotes ciliogenesis, characterized by the increase of the number of ciliated cells and cilia length (Figure 3A), which has been confirmed in cultured primary renal epithelia cells isolated from SMYD2-knockout kidneys. The activity of SMYD2 is dependent on CDK4/6-mediated phosphorylation, and SMYD2 also promotes the expression of CDK4/6 by forming a feedback loop. In addition to methylation of α-tubulin directly, SMYD2 also regulates ciliogenesis by regulating the expression of ciliary protein IFT20 at the transcriptional level [12].

The methylation of α-tubulin is mediated by SETD2, and its acetylation can occur at the same lysine 40 (K40) site, which raises a concern as to whether these two modifications to α-tubulin occur at the same time, and as to what the outcomes are of these modifications to α-tubulin stability and ciliogenesis. The fluorescence intensity of trimethyl- and acetyl-specific antibodies of α-tubulin reveals an inverse relationship between the acetylation and trimethylation of α-tubulin at K40, suggesting a possibility that acetylation and methylation of microtubules are reciprocal marks, and may function in an opposed direction to regulate microtubule polymerization during mitosis [50]. The effects of SETD2-mediated α-tubulin methylation at K40 on ciliogenesis need to be further investigated.

In addition, tubulin can be polyglutamylated. Glutamylation is an addition of a side-chain peptide composed of glutamic acids (Es) to the γ-carboxyl group of an E in the protein primary sequence [70]. Microtubule polyglutamylation is a reversible process catalyzed by tubulin tyrosine ligase-like glutamylases (TTLL) and tubulin deglutamylases that belong to the cytosolic carboxypeptidase (CCP) family (Figure 2). Tubulin polyglutamylation adds strings of glutamines near the C-terminus of either α- or β- tubulin, which generates multiple negative charges in regions of the tubulin dimer that face the microtubule surface, regulating the interaction of other proteins with microtubules [71]. The proper level of glutamylation of tubulin is essential for the function of the cilia, especially in motile cilia. Both hypo- and hyperglutamylation of tubulin will cause motility defects in motile cilia.

Tubulin glycylation, which adds glycine residues to tubulin, is very similar to glutamylation, and is catalyzed by enzymes of the tubulin tyrosine ligase-like (TTLL) family. However, there is no known deglycylase to reverse this modification [72]. Glycylation of tubulin was first thought to be a modification enriched in motile cilia or flagella, and plays a role in stabilizing the axoneme [73]. The role of tubulin glycylation in the regulation of primary cilia biogenesis has been reported recently, which has been confirmed by the depletion of glycylation-catalyzing enzymes [74]. There are three TTLLs involved in tubulin glycylation in mammals: two enzymes—TTLL3 and TTLL8—function as the initiating glycylases to add the first glycine residues to tubulin, whereas TTLL10 contributes to the elongation of glycine chains and the formation of polyglycylation [75]. However, the mechanism of epigenetic regulation on TTLLs and CCP is as yet unclear.

### 3.2. Epigenetic Mechanisms Mediate Ciliary Gene Expression in Ciliogenesis

In addition to tubulin modifications, epigenetic regulators also regulate ciliogenesis by affecting the expression of ciliary genes. We found that SMYD2 can regulate the expression of IFT20 at the transcriptional level, confirmed by ChIP assay [12]. EZH2 encodes the methyltransferase unit of polycomb repressive complex 2 (PRC2), and exhibits its role in driving metastatic melanoma formation by mediating primary cilia deconstruction through its canonical histone methylation mark H3K27me3 on the cilia loci, and inhibiting the expression of diverse ciliary genes [76] (Figure 3B). In addition, the bromodomain protein ANCCA/ATAD2 and the histone methyltransferase MLL1 regulate the transcription of the genes of the kinesin family, encoding the motor proteins for IFT B complex [77]. Furthermore, the lysines K346/348 of KIF3A were found to be acetylated in a proteomic analysis [78]; however, the function of the acetylation of KIF3A remains unknown.

### 3.3. Epigenetic Mechanisms Mediate the Modification of Other Cytoskeleton Proteins in Ciliogenesis

Cilia assembly and disassembly are tightly coupled with the actin cytoskeleton’s dynamics [51,79]. The polymerization of the actin cytoskeleton, F-actin branching, and formation of stress fibers inhibit primary cilium formation, whereas depolymerization or depletion of actin enhances ciliogenesis [80,81]. One of the epigenetic regulators—KDM3A—as we mentioned previously, has been reported to regulate cilia biogenesis, through both transcriptional regulation of the actin gene expression, and directly binding with actin. The interaction between KDM3A and the actin proteins may locally form an actin gate at the ciliary base, which then regulates the entry of IFTs. In the absence of KDM3A, access limitation to cilia is diminished, and disrupts the balance and/or kinetics of IFTs [51] (Figure 3B).

## 4. Crosstalk between the Cell Cycle and Ciliogenesis

With the knowledge of the process of ciliogenesis, from initiation and elongation to resorption, it is necessary to understand the crosstalk between the cell cycle and ciliogenesis. In general, the cilia assembly and disassembly cycle is closely associated with the cell cycle, as described above, which suggests that the cilia biogenesis process can be passively regulated by cell cycle progression. However, several studies have provided some new clues about the relationship between the primary cilia and the cell cycle. Firstly, studies have indicated that by the inhibition of several negative regulators of ciliogenesis, cilia formation was maintained in proliferating cells and the cell cycle was arrested, while overexpression of these factors inhibited cilia formation, even in serum-starved cells [82]. It has been reported that trichoplein complexed with Aurora A (Aur A) blocks aberrant primary cilia assembly in proliferating cells. Overexpression of trichoplein inhibited primary cilia assembly in serum-starved cells, whereas knockdown of trichoplein induced primary cilia assembly, even in serum-contained medium, and induced G_0_/G_1_ arrest [83], suggesting the independence of ciliogenesis from the cell cycle. The following findings also indicate that ciliogenesis can function as a regulator of the cell cycle and cell proliferation: (1) defects of the cilia or lack of cilia cause cellular proliferation abnormalities and diseases; (2) primary cilia sense urine flow, and are essential for the maintenance of the epithelial architecture in the kidneys; and (3) defects of primary cilia cause cystic kidney disease characterized by epithelial abnormalities, suggesting the roles of primary cilia in the regulation of the cell cycle and cell proliferation.

### 4.1. Canonical View of Ciliogenesis and the Cell Cycle

Primary cilia have now been recognized as sensors in response to extracellular chemical and mechanical stimuli. The canonical view is that the cilia assembly and disassembly cycle is dependent on the cell cycle, in that cilia appear when cells exit from cell cycle M/G_1_ into the G_0_ phase, disappear when cells re-enter the cell cycle, and reappear post-cytokinesis [11]. The cilia disassembly process is switched on upon G_1_/S entry, which can be regulated by the interaction between the prometastatic scaffolding proteins HEF1/Cas-L/NEDD9 and the oncogenic serine/threonine kinase Aurora A (Aur A) at the basal body of the cilia. Aur A also can regulate cilia disassembly through HDAC6-mediated deacetylation of α-tubulin, and this process can be regulated by HEF1 [84]. In addition, Aur A can regulate the cilia disassembly process in the G_1_ phase through affecting the localization of the IFT–B complex protein IFT88. Aur A was found to be highly activated in ovarian cancers. Inhibition of Aur A not only induced ovarian cancer cell cycle arrest, but also restored cilia assembly in those cells [85]. It has also been reported that von Hippel–Lindau (VHL) and GSK3β—which are dysregulated in clear cell renal cell carcinoma and ovarian cancers—are required for primary cilium maintenance, and dysregulations of these proteins result in defects in cilia formation, and promote cyst growth in VHL diseases [86]. Thus, cilia loss and cell proliferation are common phenomena across different cancers, including pancreatic cancers, human primary melanoma, ovarian cancer, and renal cancers [84,85].

### 4.2. Cilia Play a Predominant Role in Cell Cycle and Cell Proliferation Regulation

Growing evidence supports a role for cilia in cell cycle regulation. First, it has been reported that the deletion of Tg737—a murine homolog of IFT88—results in shorter cilia than normal in murine kidney cells [87], identifying IFT88/Tg737 as a key factor for primary cilia assembly in mammals. Importantly, mice with defects in Tg737 die shortly after birth from polycystic kidney disease, supporting the notion that primary cilia play a critical role in the kidneys, and that defects in cilia assembly can lead to polycystic kidney disease [87]. In addition, it has been found that when IFT88 is overexpressed, it prevents the G_1_/S transition, and when depleted by RNA interference (RNAi), it promotes cell cycle progression to the S, G_2_, and M phases [88]. One of the mechanisms of IFT88 in the regulation of the cell cycle is through its interaction with Che-1—a key regulator of S phase entry—to regulate Che-1 localization and activity, eventually modulating the binding of Che-1 to Rb and regulating the cell cycle. Second, it has been reported that IFT27—another IFT–B complex protein—is also associated with the cell cycle. Cells with depletion of IFT27 by RNAi not only failed to form flagella but were also defective in cytokinesis. The molecular function of IFT27 in cell cycle progression is still unknown [89]. Third, it has been found that depletion of nuclear distribution gene E homolog 1 (Nde1)—a negative regulatory factor of cilia length—shows longer cilia and a delay in cell cycle re-entry, suggesting a correlation of ciliary length with cell cycle progression [90]. In addition, more evidence for cilia in the regulation of cell cycle progression is provided by the studies of cilia-dependent signaling pathways.

### 4.3. Epigenetic Mechanisms Regulate Crosstalk between the Cell Cycle and Cilia Biogenesis

We have discussed the role of epigenetic regulators in the regulation of gene expression and protein function related to the cell cycle and ciliogenesis separately. In this section, we summarize the known epigenetic regulators that are involved in the connections of these two processes (Table 1). In brief, histone/lysine methyltransferase SMYD2 could promote cell cycle progression by regulating the expression of CDK4/6 and chromatin status through the methylation of histone H3 at lysine 4 and lysine 36 [12,33,91]. SMYD2 also regulates ciliogenesis by regulating the transcription of IFT20 and the methylation of α-tubulin at lysine K394 [12]. Another histone/lysine methyltransferase—SETD2—methylates histone H3K36 and α-tubulin at K40 to regulate the cell cycle, and potentially regulates ciliogenesis synergistically [48,49,50]. The third histone/lysine methyltransferase—EZH2—exerts its function in the regulation of the cell cycle and ciliogenesis through the methylation of histone H3K27 to regulate the expression of genes associated with these two processes [76,92]. Histone demethylase KDM3A regulates both the cell cycle and ciliogenesis through targeting actin to either bind with its promoter to regulate its expression or directly bind to the actin cytoskeleton to form a complex that functions as a ciliary actin gate [51]. HDAC6 induces cilia disassembly by directly deacetylating α-tubulin at K40 in an Aur-A-dependent manner, while it regulates the cell cycle through an indirect signaling pathway, relying on TGF-β–SMAD2-mediated p21 expression [14,24].

## 5. Cilia-Dependent Signaling Pathways in Cell Cycle Regulation, and the Association of Epigenetic Mechanisms with These Signaling Pathways

Cilia also function as regulatory switches to control diverse signaling pathways, including Hedgehog-, Wnt-, PDGFR-, Notch-, TGF-β-, mTOR-, and GPCR-associated signaling. All of these signaling pathways play crucial roles in various cellular processes, such as in organ and embryonic development, cardiac function, planar cell polarity, transactivation, differentiation, cell cycle progression, apoptosis, tissue homeostasis, and the immune response. Growing evidence demonstrates that these signaling pathways can be regulated by epigenetic mechanisms in human diseases.

### 5.1. Cilia-Dependent Signaling Pathways in Cell Cycle Regulation

Hedgehog (Hh) signaling plays an essential role in many aspects of vertebrate embryonic development and tissue homeostasis [93]. The core components of the Hh pathway include the vertebrate Hh receptor Patched1 (Ptch1), the obligate transducer of the pathway Smoothened (Smo), and the Gli transcription factors that act as both activators and repressors to control target gene transcription [94]. Deregulation of the Hh pathway is associated with transformation and tumorigenesis, as well as drug resistance, in a multitude of cancers, highlighting the importance of the pathway and its regulation [95]. Primary cilia play an essential role in the activation of Hh signaling. In the absence of the Hh ligand, Ptch1 is enriched on the ciliary membrane, where Smo is barely detectable, and full-length Gli proteins traffic to the ciliary tip and back to the cytoplasm before being cleaved to their repressor form, which actively shuts down Hh target gene transcription in the nucleus [96]. Upon stimulation with Hh ligands (Shh, Ihh, or Dhh), Ptch1 binds the ligand and shuttles out of the cilia, and Smo is enriched in the cilium and is subsequently activated. Activated Smo promotes the cleavage of full-length Gli proteins, which turns on target genes [97]. Hh signaling could promote the transcription of cyclin E and cyclin D, which provide a direct connection between Hh signaling and cell cycle regulation, and a mechanism by which the deregulation of Hh signaling promotes tumorigenesis [98]. In addition, Hh may have a more immediate influence on the cell cycle through regulation of the Patched (Ptch)-mediated subcellular localization of the M-phase-promoting factor (MPF), in that Ptch1 interacts with MPF to regulate its activity [99]. Thus, discovering the regulators of the cell cycle and ciliogenesis associated with the Hh signaling pathway should further our understanding of the crosstalk of these processes, and identify therapeutic targets of interest.

Wnt/β-catenin signaling is another key signaling cascade, essential for both embryonic development and tissue homeostasis in adulthood. Central to this pathway is a multiprotein scaffold composed of adenomatous polyposis coli (*APC*), glycogen synthase kinase (*GSK*)–3β, axin, and the transcriptional cofactor β-catenin. Binding of the Wnt ligands to their receptors stabilizes β-catenin and results in its translocation to the nucleus, where it binds members of the T-cell factor (Tcf)/lymphoid-enhancing factor (Lef) family of transcription factors, and induces the expression of target genes such as *CMYC* and *CCND1* [100]. It has been reported that the primary cilium dampens canonical Wnt signaling through a unique spatial mechanism involving compartmentalization of signaling components. To maintain the appropriate signal strength of Wnt/β-Catenin signaling, cilia keep β-catenin away from the nucleus to limit β-catenin-mediated expression of intraflagellar transport (IFT) and Jouberin (Jbn). In addition, Wnt signaling also regulates ciliogenesis. Wnt5a bound with receptors triggers casein kinase 1 epsilon (CK1ε) activation to induce Dishevelled 2 (Dvl2) phosphorylation. Phosphorylated Dvl2 can bind with Plk1 to inhibit Smad3-dependent HEF1 degradation, which enhances HEF1-dependent cilia disassembly through Aur A and, in turn, cell cycle progression [101]. Furthermore, when β-catenin is hyperactivated, the expression of cyclin D1 and c-Myc is upregulated, which promotes G_1_ progression [102].

Platelet-derived growth factor (PDGF) and its receptors (PDGFRs) play pivotal roles in cell proliferation, survival, and migration, as well as embryonic development and tissue development. The PDGF family consists of four members—PDGF-A, -B, -C, and –D—forming disulfide-linked homodimers PDGF-AA, BB, CC, and DD, and the heterodimer PDGF-AB [103], which exert their action via binding to and dimerization of two receptor tyrosine kinases—PDGF α-receptor (PDGFR-α) and β-receptor (PDGFR-β) [104]. PDGFR-α is upregulated during ciliogenesis, and its ciliary localization is required for signaling activation mediated by PDGF-AA [105]. PDGF-AA functions as a “competent factor” that stimulates the transit of the cell cycle beyond the G_1_/S checkpoint [106]. In addition, the interaction of IFT20 with E3 ubiquitin ligases c-Cbl and Cbl-b is required for Cbl-mediated ubiquitination and internalization of PDGFR-α signaling [107]. In the presence of IFT20, PDGF-AA stimulation induces c-Cbl enrichment in the cilium, and results in subsequent ubiquitination and internalization of PDGF receptors, avoiding the overactivation of PDGF signaling. While in the absence of IFT20, PDGFR-α localizes to the plasma membrane, and is constitutively activated after ligand stimulation due to the lack of ubiquitination mediated by c-Cbl and Cbl-b.

Other cilia-dependent signaling pathways—such as Notch signaling, and transforming growth factor beta (TGF-β)—are also associated with the cell cycle. Notch signaling plays a key role in various aspects of patterning and cell fate choices in neurogenesis and the maintenance of adult tissue growth and development. Studies suggest that some Notch signaling may depend on the primary cilium for signal transduction [108]. The Notch 3 receptor has been shown to localize in the ciliary membrane [109]. In addition, Notch signaling could regulate subcellular localization of the Shh receptor Patched1 and the key effector Smoothened to the primary cilia, thereby influencing the cell fates of neural progenitors [110]. Transforming growth factor beta (TGF-β) plays major roles in bone development and maintenance metabolism by regulating cellular proliferation, differentiation, matrix deposition, and cell migration. The TGF-β/SMAD2/3 axis plays an important role in renal fibrosis in the pathogenesis of polycystic kidney diseases. Recent studies have demonstrated that TGF-β1 and TGF-β2 receptors are localized within and around the primary cilium in embryonic fibroblasts [111]. Stimulation with TGF-β increases the activation of SMAD2/3 and ERK1/2 at the ciliary base [111].

### 5.2. The Association of Epigenetic Mechanisms with Cilia-Dependent Signaling Pathways

Epigenetic regulators have been shown to be critical factors in the determination of cell signaling responses in temporal and spatial regulation, which has led to a greater appreciation of how the epigenome interplays with cell signaling to influence development and disease progression. Aberrant activation of cilia-dependent signaling pathways occurs in a wide range of human diseases. The impact of epigenetic regulation of cilia-dependent signaling pathways is becoming increasingly clear. Epigenetic control of cilia-dependent signaling pathways may take place through DNA methylation and histone acetylation/methylation, regulating the production of ligands and the expression of pathway target genes, followed by the epigenetic regulation of the extra- and intracellular pathway members. The central player of the cilia-dependent signaling pathways may also interplay with the epigenome by recruiting epigenetic regulators to the target gene regulatory elements. In this section, we summarize the known epigenetic regulations on the cilia-dependent signaling pathways described above.

Aberrant production of Hh ligands (Shh, Ihh, or Dhh) is found in many cancers and defective diseases, and DNA methyltransferase (DNMT)-mediated hypomethylation or hypermethylation of these gene promoters was identified as the main reason. DNMTs also regulate other Hh signaling components by the same mechanism, including Ptch1, Smo, and other co-receptors. In addition to DNMTs, micro-RNAs (miRNAs) provide alternative mechanisms to regulate these receptors as well. It has been reported that miR-125b, miR-324-5p, and miR-326 target and suppress Smo through binding to the 3′-UTR of the SMO gene. Similarly, miRNAs are the main regulators of the GLI proteins, which function as the terminal effectors of Hh signaling [112]. For example, Gli1 is suppressed by miR-324-5p-mediated 3′-UTR regulation [113]. DNMT-mediated promoter methylation and miRNA-mediated regulation are also ubiquitous mechanisms in regulating most components of the Wnt signaling pathway, including ligands, receptors, and co-receptors [112]. WNT5A promoter methylation was frequently detected in colorectal cancer, which could be reversed by genetic demethylation [112]. In addition, WNT5A is also regulated by miR-374a-mediated suppression by targeting its 3′-UTR to activate the β-catenin pathway. It should be noted that miR-374a also targets Wnt inhibitory factor-1 (WIF1)—a secreted antagonist of the Wnt pathway [114]. Since IFT20 plays an essential role in maintaining the appropriate strength of PDGF signaling, and it could be regulated transcriptionally by SMYD2, SMYD2 functions as one of the epigenetic factors that regulate PDGF signaling. In addition to this, PDGFR-β was reported to be regulated by acetyltransferase-p300-mediated H3K27 acetylation on its promoter [115]. DNA methylation on the promoters of Notch ligands JAG1 and JAG2 contributes to their expression, and loss of this modification increases the expression and activates Notch signaling [116]. SIRT1 functions as a negative modulator of Notch signaling in endothelial cells, in which Notch could be destabilized by SIRT1-mediated deacetylation [117]. In addition, the transcriptional regulation of Notch activation can also be controlled by the commitment of miRNAs—such as miR-34c—which could target multiple components of the Notch signaling pathway, including Notch1, Notch2, and Jag1 [118]. HATs and HDACs are involved in the regulation of TGF-β signaling. p300 has been reported to acetylate SMAD2 and SMAD3 at specific lysine residues and enhance their capacity to bind DNA, while HDACs act as transcriptional co-repressors, limiting TGF-β target gene expression [119]. In addition, the central players of the TGF-β pathway—R-SMADs—interplay with the epigenome through recruiting epigenetic regulators—such as chromatin remodelers, histone modifiers, DNA modifiers, chromatin readers, and lncRNAs—to the target gene regulatory elements. In some cases, epigenetic regulators are among the target genes, and are induced by R-SMADs [120].

## 6. Therapeutic Targets of Cell-Cycle- and Ciliogenesis-Associated Epigenetic Regulators

Increasing evidence exhibits the roles of epigenetic regulators in regulating the cell cycle and ciliogenesis during organ development, and in many proliferative diseases (such as cancers) and cilia-deficient diseases (termed as ciliopathies) [33,66,76]. Thus, targeting abnormal epigenetic regulators might be an advanced therapeutic strategy.

The role of the histone methyltransferase EZH2 in driving transformation from benign to malignant melanoma has been associated with the regulation of both the cell cycle and ciliogenesis. On one hand, EZH2 suppresses the expression of ciliary proteins through its repressive histone mark H3K27me3, by binding to the promoters of many ciliary genes, including WDR19, IFT81, FUZ, etc. On the other hand, loss of cilia results in the inappropriate activation of cilia-dependent signaling. In A375 human melanoma cells, the enhanced WNT/β-catenin activity and nuclear accumulation is accompanied by loss of cilia, due to the absence of cilia-mediated spatial segregation. The involvement of EZH2 in this process was evidenced by the findings that increased EZH enhances WNT/β-catenin signaling in benign melanocytic cells, and inhibition of EZH2 with GSK503 induces cilia assembly in most melanoma cell lines. Blockade of EZH induces cilia assembly, which could be reversed by silencing ciliary genes [76]. The EZH2 inhibitor GSK503 is a small molecule, which exerts its inhibition by competition with S-adenosylmethionine (SAM), the donor of the methyl group. There are several other EZH2 inhibitors—such as DZNep, EPZ005687, EI1, GSK126, UNC1999, GSK503, and EPZ-6438—which are all small molecules and function as SAM-competitive inhibitors. DZNep is one of the purine nucleoside analogs, which has been tested in the treatment of hematological malignancies and autoimmune diseases. EPZ005687 is a selective inhibitor of EZH2 rather than EZH1. EI1 mainly regulates and activates EZH2 target genes via the removal of repressive histone mark H3K27 methylation. GSK126 has been tested in a live failure murine model, in which it decreased inflammation response through the reduction of H3K27 methylation. UNC1999 functions similarly to GSK126. GSK503 has been tested in a human melanoma murine model. EPZ-6438 is an improved product of EPZ005687. Among those inhibitors, tazemetostat (EPZ-6438) has been approved for the treatment of epithelioid sarcoma [121]. Whether those EZH2 inhibitors also affect ciliogenesis in different disease models needs to be further investigated.

Histone methyltransferase SMYD2 is involved in both cell cycle regulation and ciliogenesis. Blockade of SMYD2-induced cell cycle arrest at the G_1_/S phase was observed in breast cancer cells and *Pkd1*-mutant cystic renal cells [33,91]. In addition, SMYD2 regulates cilia biogenesis not only through regulating the expression of IFT20, but also by affecting microtubule stability via the methylation of α-tubulin. The cell cycle regulators CDK4 and CDK6 are involved in this process. Inhibition of either SMYD2 or CDK4/6 would restore the ciliation of cystic renal epithelial cells and breast cancer cells [12]. The SMYD2 inhibitor AZ505 exerts its function by competitively binding to the substrate-binding groove of SMYD2. Another selective SMYD2 inhibitor—LLY-507—also binds to the substrate–peptide-binding pocket. The inhibitory effect of LLY-507 on the inhibition of cell proliferation has been observed in multiple tumor cell lines. The SMYD2 inhibitor A-893 has higher inhibitory activity on SMYD2 than AZ505. BAY-598 acts as a substrate-competitive inhibitor of SMYD2 [122]. CDK4/6 mediate cell cycle progression, mainly through the phosphorylation of tumor repressor Rb, in which the phosphorylation of Rb can be regulated by SMYD2-induced methylation of Rb. Phosphorylation-induced inactivated Rb functions as an oncogene. Inhibition of CDK4/6 induces reactivation of its suppressor function, and results in cell cycle arrest at the middle of the G_1_ phase, and subsequent tumor repression. CDK4/6 inhibition has been mainly targeted towards the treatment of breast cancers. Three CDK4/6 inhibitors (palbociclib, ribociclib, and abemaciclib) have been approved to be applied to different subclasses of breast cancer. Accordingly, it has been confirmed that abemaciclib treatment could inhibit cell proliferation and restore cilia formation, either inducing ciliation in cilia-bare breast cancer cells or increasing cilia length [12]. The effects of other SMYD2 inhibitors in affecting cilia biogenesis are worthy of being determined in different diseases, such as PKD.

The cellular ciliation state is determined by both cilia assembly and disassembly processes. The HEF1–Aur A–HDAC6 axis is the main path to regulate cilia disassembly. Aur-A-activated HDAC6 deacetylates α-tubulin at K40, resulting in destabilization of microtubules, and induces cilia disassembly and cell cycle re-entry. Thus, HDAC6 inhibition should reverse cilia disassembly and reinforce the cilia, leading to cell cycle suspension. The deacetylation function of HDAC family members requires a zinc ion for their activity, and shares conserved residues in their active sites. Due to the similarity of the conserved catalytic sites of HDACs, pan-HDAC inhibitors have been used in clinical trials for the treatment of multiple myeloma and other hematological tumors. Several HDAC inhibitors have been approved by FDA for treatments, such as vorinostat (SAHA) for clinical use in cutaneous T-cell lymphoma (CTCL). However, because of the side effects of pan-HDACs, the specific and selective HDAC6 inhibitors have been synthesized, which all belong to the class of hydroxamic acids. Tubacin and tubastatin are small molecular inhibitors of HDAC6. Tubacin has been used in multiple myeloma and lymphoma. Inhibition of HDAC6 by tubacin increases the acetylation of α-tubulin, which prevents cilia disassembly [14]. In addition, because the activity of HDAC6 is dependent on its phosphorylation mediated by Aur A, inhibition of Aur A with inhibitors should induce a similar effect as does the inhibition of HDAC6 on ciliogenesis and cell cycle progression. So far, more than 20 Aurora kinase inhibitors have been developed. The challenge for the clinical use of these inhibitors is their specificity. The Aurora kinase inhibitor ZM447439 targets both Aurora A and B, while VX-680 targets all three kinases (Aurora A, B, and C). It is not clear whether Hesperadin also targets Aurora A and C, even though it mainly targets Aurora B. PHA-680632, which was tested to protect cilia from disassembly in cell assays, also targets all three kinases, with differences of IC_50_s [14].

EZH2 and SMYD2 mainly exert their regulatory functions in cilia assembly, and inhibition of either of them promotes ciliogenesis and slows down the cell cycle, while Aur A and HDAC6 promote cilia disassembly and cell cycle progression, and inhibition of Aur A and HDAC6 decreases cilia disassembly and delays the cell cycle, leading to the same outcomes in cancers and ciliopathies.

In addition to their roles in regulating the cell cycle and cilia components, several epigenetic regulators have been shown to directly control certain aspects of cilia-dependent signaling pathways, which might plausibly offer a route to successfully targeting those pathways. Epigenetic modulators were virtually screened for their efficiency in inhibiting key regulators of the SHH pathway—including sonic hedgehog, Smoothened, and Gli—using a pharmacological approach. Histone/lysine methyltransferase inhibitors—such as EZH2 inhibitors, HDAC inhibitors, and UNC 1215 (L3MBTL3 antagonist)—exhibited a multiple-targeted approach in modulating Hh signaling [123]. Several research groups have studied HDAC and HAT inhibitors in other cilia-dependent signaling pathways, and novel inhibitors that target histone-modifying enzymes are being developed and tested. It is noteworthy that many of the recently developed inhibitors that target aberrantly expressed epigenetic regulators are being derived from naturally occurring substances, such as curcumin, embelin, garcinol, and polyphenols in green tea [119]. In sum, growing evidence indicates that the components of cilia-dependent signaling pathways are epigenetically regulated, and are actively involved in the pathogenesis of various human malignancies. Further investigations are necessary in order to translate the current epigenetic discoveries into useful therapeutic tools for diseases associated with cilia-dependent signaling.

## 7. Conclusions and Perspectives

Increasing evidence supports the contributions of epigenetic regulators in the pathogenesis of cancers and ciliopathies, which are caused by cell cycle dysregulation, cilia defects, or both. In general, the cell cycle and cilia biogenesis are finely regulated by synergistic or independent mechanisms. The cell cycle communicates with the cilia assembly/disassembly cycle through multifaceted means, such as the common components of centrosome and ciliary proteins, or cilia-dependent signaling pathways—including Hedgehog signaling, Wnt signaling, and Notch signaling, among others. Epigenetic mechanisms play critical roles in the regulation of the cell cycle and cilia biogenesis, as well as their crosstalk. Epigenetic regulators are involved in all stages of cell cycle progression and cilia biogenesis through: (1) direct modifications of key cell cycle regulators, including CDKs and cyclins; (2) modifications of histone marks to regulate the expression of genes necessary for cell cycle progression and cilia biogenesis, via changes to chromatin structures; and (3) regulation of the cell-cycle- and ciliogenesis-associated cytoskeleton network via modifications to actin and microtubules. Importantly, some epigenetic regulators are the mediators of the crosstalk between the cell cycle and ciliogenesis in organ development and diseases. Dysregulation of epigenetic regulators is one of the common characteristics of cancers and ciliopathies, and could serve as a therapeutic target for both. For example, SMYD2 was upregulated in triple-negative breast cancer and ADPKD—both of which are associated with cilia dysfunction—and treatment with SMYD2 inhibitors suppresses tumor growth and delays cyst growth in animal models [33,91]. SETD2 methylates α-tubulin at K40, and deficiency of SETD2 causes cytokinesis defects and cell cycle delay; however, whether SETD2 regulates cilia biogenesis via the methylation of α-tubulin at K40 needs to be further investigated [50]. With the ever-growing technological advancements in the field, more epigenetic mechanisms associated with cell cycle regulation and cilia biogenesis will no doubt be uncovered, and this will facilitate the discovery of epigenetic drugs in the treatment of human diseases.

## Figures and Tables

**Table 1:** The dual roles of epigenetic regulators in cell cycle and ciliogenesis regulation.

Epigenetic Regulators	Cell-Cycle-Related Target(s)	Ciliary Target(s)	References
SMYD2	CDK4/6, H3K4 me3 and H3K36 me3	IFT20, α-tubulin	[12,33,92]
SETD2	H3K36me3	α-tubulin	[48,49,50]
EZH2	H3K27me3	WDR19, IFT81, FUZ, etc.	[76,93]
KDM3A	Actin	Actin at basal body	[51]
HDAC6	p21	α-tubulin	[14,24]
